# [Retracted] Reduced expression levels of the death-associated protein kinase and E-cadherin are correlated with the development of esophageal squamous cell carcinoma

**DOI:** 10.3892/etm.2026.13250

**Published:** 2026-07-27

**Authors:** Jianwen Zhai, Xiaogang Yang, Yanli Zhang, Qingbin Qi, Jigang Hu, Qiaomei Wang

Exp Ther Med 5:972–976, 2013; DOI: 10.3892/etm.2013.916

Following the publication of this paper, it was drawn to the Editor’s attention by a concerned reader that certain of the western blot data featured in Fig. 2 on p. 974 were strikingly similar to data that had already appeared in different form in Fig. 3C of another article written by different authors at different research institutes in the journal *Proceedings of the National Academy of Sciences,* albeit with some horizontal and vertical resizing of the bands concerned.

In view of the fact that the abovementioned data had already apparently been published before this article was submitted to *Experimental and Therapeutic Medicine*, the Editor has decided that this paper should be retracted from the Journal. The authors were asked for an explanation to account for these concerns, but the Editorial Office did not receive a reply. The Editor apologizes to the readership for any inconvenience caused.

